# When to Transfer Embryos if There Is Only 1 or 2?

**DOI:** 10.3390/life13020417

**Published:** 2023-02-02

**Authors:** Martin Stimpfel, Nina Jancar, Helena Ban-Frangez, Eda Vrtacnik-Bokal

**Affiliations:** 1 Department of Human Reproduction, Division of Obstetrics and Gynecology, University Medical Centre Ljubljana, 1000 Ljubljana, Slovenia; 2 Faculty of Medicine, University of Ljubljana, 1000 Ljubljana, Slovenia

**Keywords:** embryo transfer, blastocyst, cleavage stage embryo, birth rate, embryo development

## Abstract

The latest reports suggest that it is better to transfer embryos to the uterus on day five of preimplantation development compared to other days of development, but it is not clear if this stands when there are only one-two embryos obtained in the cycle. Therefore, to address this issue, we performed a retrospective study of such cycles. Our study included all of the stimulated IVF/ICSI cycles performed at our institution in the period between 1 January 2004 and 31 December 2018 in which one-two embryos were obtained in the IVF/ICSI cycle and met our inclusion criteria, and we compared the data between day three and day five embryo transfer (ET). The analysis revealed that the day three ET group of patients was significantly older (*p* < 0.001), were administered a significantly higher dose of gonadotrophins (*p* = 0.015), and retrieved a lower mean number of aspirated oocytes per cycle (*p* < 0.001) and lower mean number of embryos (*p* < 0.001). The birth rate per ET was significantly higher in the day five ET group (*p* = 0.045) and further analysis indicated that this could be due the trend observed in a group of patients under 36 years old, while in older patients there was no such difference. To conclude, our retrospective study indicates that it might be better to perform ET on day five instead of day three when there are only one-two embryos obtained in the cycle, but probably only when patients are under 36 years old.

## 1. Introduction

The debate concerning which developmental stage of embryos or the day of preimplantation development is most optimal for embryo transfer (ET) to the uterus is still ongoing. Many studies and even meta-analyses have been conducted, but the conclusions are still too often contradictory. For instance, a retrospective study from 2017, which was combined with meta-analysis, showed improved cumulative pregnancy and live birth rates for day 5/6 ETs compared to day 2/3 ETs, although the difference was not significant [1]. Nevertheless, this study suggested that day 5/6 ET is more cost-effective and time efficient. Similar was suggested by Cochrane meta-analysis [2] where higher birth and clinical pregnancy rates after fresh blastocyst ET compared to cleavage stage ET were observed. Furthermore, this study suggested that cumulative pregnancy, multiple pregnancies, and miscarriages are similar no matter on which day ET is performed. On the contrary, another meta-analysis from 2017 [3] suggested there is no significant difference in live birth/ongoing pregnancy, clinical pregnancy, cumulative pregnancy and miscarriages between different ET days. Furthermore, a meta-analysis by Alviggi et al. [4] suggested a higher incidence of preterm and very preterm births after fresh blastocyst transfer compared to cleavage-stage embryo transfer, although there were fewer small for gestational age deliveries after fresh blastocyst transfer. Similar conclusions were drawn in the latest meta-analyses. For example, it was suggested that blastocyst transfer is associated with a higher risk for large for gestational age and also with preterm birth [5,6], and that stands for fresh and frozen ETs [5]. Furthermore, as it was suggested in the previously mentioned meta-analyses, the latest data also suggest that single blastocyst transfer results in higher clinical and ongoing pregnancy rates and delivery rates, but there is no difference in the miscarriage rate, multiple pregnancy rate and ectopic pregnancy rate [7]. On the other hand, when cleavage stage embryo transfer is performed, there seems to be more cryopreserved embryos at this stage, as they are at the blastocyst stage [7]. In summary, these meta-analyses suggest that blastocyst transfer is more successful than cleavage stage ET, although these studies do not take into consideration whether there is any difference regarding the number of retrieved oocytes at aspiration or the number of obtained embryos. For instance, it was recently shown that blastocyst transfer leads to higher clinical pregnancy rates compared to cleavage stage embryo ET if six or more zygotes are obtained, otherwise there is no difference [7]. While this is very informative, it still does not answer the question of when it is better to transfer embryos if there is only one or two obtained. This is important from the perspective that blastocyst transfer enables the selection of embryos that seem to have better potential for further development, but this is possible only when a higher number of embryos are obtained. If there is only one or two embryos, additional selection is not possible, therefore the question arose if it is better to put embryos back into in vivo conditions into the uterus as soon as possible or there is no harm if they are cultured for an extended time in vitro. As we face this dilemma in clinical practice frequently, and the data in literature is scarce, we performed this retrospective analysis of cycles performed at our institution where only one or two embryos were obtained. To determine which day of ET is more efficient in our conditions, we begin with analyzing the correlations of the variables and then divide the cycles according to the day of ET and compare the outcomes between these two groups in terms of oocytes, embryos, pregnancy and live birth rate, gestational age and birthweight.

## 2. Materials and Methods

This retrospective study was carried out at the Department of Human reproduction, Division of Obstetrics and Gynecology, University Medical Centre, Ljubljana. At the beginning, we included all of the stimulated IVF/ICSI cycles performed in the period between 1 January 2004 and 31 December 2018 where only 1 or most 2 embryos were obtained after conventional IVF or ICSI procedure. Then, to obtain the IVF/ICSI cycles to perform the statistical analysis, we excluded: cycles with oocyte donation, cycles with oocyte thawing, cycles where testicular sperm was used for ICSI, cycles with preimplantation genetic testing, cycles with ET on cleavage day 2 and freeze-all cycles. Most of the included patients had a low response (73.4% of them had 5 or less retrieved oocytes). First, we statistically analyzed all of the cycles together and then further divided the cycles in two groups: cycles with embryo transfer (ET) at cleavage stage on day 3 (day 3 ET group) and cycles with ET at blastocyst stage on day 5 (day 5 ET group). As the correlation analysis showed that the age of the patients is strongly correlated with the ET outcome and the day 3 ET group and day 5 ET group had significantly different patient mean ages, we further analyzed the data for the most important variables according to the age of the patients. Therefore, we separately analyzed the data for the group of patients less than 36 years old and for the group of patients older than 35 years (up to 43 years). 

This was a register-based study in which all of the participants signed individual personal approval and permission before starting the treatment and did not have to be notified in the Ethics Committee according to Slovene law, (Personal Data Protection Act, Official Gazette of the Republic of Slovenia No 94/07, 2004). Additionally, by our law, we are obligated to collect the data concerning assisted reproduction procedures and monitor the success rates (Healthcare Databases Act, Official Gazette of the Republic of Slovenia No 65/00, 2000; No 47/15, 2015; 31/18, 2018).

The data were analyzed with the Statistical Package for the Social Sciences software (SPSS, version 21, IBM, Armonk, NY, USA). To determine the correlations between the variables, a Pearsons correlation test was performed. To determine the differences between the groups, the obtained data were analyzed using the Pearson’s chi-square test, Fisher’s exact test and Student *t*-test. For a statistically significant difference, a *p* value of less than 0.05 was determined.

## 3. Results

We retrospectively analyzed the outcome of IVF/ICSI cycles where there were only one or two embryos obtained and compared the outcomes regarding to the day of embryo transfer, on day three at the cleavage stage or on day five at the blastocyst stage, respectively. Altogether, 2658 stimulated IVF/ICSI cycles met our inclusion criteria. The correlation analysis (Table 1) revealed several correlations depending on the day of embryo transfer. Of the main outcomes, the day of embryo transfer was negatively correlated with the age of the patient (*p* < 0.001) and with the dose of administered gonadotrophins (*p* = 0.015), and positively correlated with the number of aspirated oocytes (*p* < 0.001), the number of embryos (*p* < 0.001) and the number of births (*p* < 0.001). Furthermore, the age of the women was revealed as a very important factor, as, among other differences, it was significantly negatively correlated (*p* < 0.001) with the pregnancy and birth rates. When only cycles where singletons were born were analyzed using correlation, there were no correlations of the gestational age and birthweight of singletons with the other variables; only gestational age was strongly correlated with birthweight (Table 2).

When we analyzed the data according to the day of embryo transfer (cleavage stage day three (day three ET group) vs. blastocyst stage day five (day five ET group), it was revealed that most of the ETs were performed at the cleavage stage (72.5%). When the outcomes of these two approaches were compared, some statistically significant differences were revealed, as already indicated by the correlation analysis. The detailed outcomes of this analysis are presented in Table 3 and Table 4. Briefly, if the most important variables are described, it was revealed that the day three ET group of patients was significantly older (36.0 ± 4.4 vs. 35.1 ± 4.5; *p* < 0.001), were administered a significantly higher dose of gonadotrophines (2228 ± 911 IE vs. 2133 ± 860 IE; *p* = 0.015), although this resulted in a significantly lower mean number of aspirated oocytes per cycle (4.3 ± 3.2 vs. 4.8 ± 2.8; *p* < 0.001), *p* = 0.002) and lower mean number of embryos (1.5 ± 0.5 vs. 1.6 ± 0.5; *p* < 0.001). Interestingly, the rate of the specific causes of infertility was mostly similar, although there were significantly more couples where both partners had a known factor of infertility in the day five ET group (39.8% vs. 45.1%; *p* = 0.012). A significant difference was also observed when the most important outcome was analyzed, while it was shown that the birth rate per ET was significantly higher when the embryos were transferred on day five (13.8% vs. 16.8%; *p* = 0.045).

As the correlation analysis showed that the age of the patients is strongly correlated with the ET outcome and the day three ET group and day five ET group had significantly different patient mean ages, we further analyzed the data for the most important variables according to the age of the patients. Therefore, we separately analyzed the data for the group of patients less than 36 years old and for the group of patients older than 35 years (up to 43 years). For the younger group, the results revealed (Table 5) that the only strong significant difference is in the number of embryos per cycles, which was higher in the day five ET group (1.5 ± 0.5 vs. 1.6 ± 0.5; *p* < 0.001). The results also indicated a weak trend for a higher rate of births per cycle in the day five ET group (18.0% vs. 22.3%; *p* = 0.075). The other tested variables (mean age of patients, mean number of aspirated oocytes, mean number of immature oocytes, mean number of transferred embryos, pregnancy rate, and twins’ rate) showed no significant difference. On the other hand, in the group of patients older than 35 years, different results were obtained. There was no significant difference in the mean number of transferred embryos, pregnancy rate, miscarriage rate, birth rate, and twins rate, although there was a significantly higher number of aspirated oocytes (3.8 ± 2.6 vs. 4.1 ± 2.4; *p* = 0.012), number of immature oocytes (0.8 ± 1.3 vs. 1.0 ± 1.3; *p* = 0.013) and number of embryos per cycle (1.5 ± 0.5 vs. 1.6 ± 0.5; *p* < 0.001) in the day five ET group. 

Additionally, we checked whether there is any difference in the cycle outcome according to the number of embryos transferred in uterus and the day of embryo transfer (Table 6). Not all of the obtained embryos were transferred in all of the cycles. In some cases, one embryo was transferred, despite two being obtained, because this was the couple’s first cycle, or the patient was young, there was a medical indication, or on the patient’s request. When cycles with the same number of transferred embryos were compared, no differences in the pregnancy, miscarriage, birth and twins rates were observed. As expected, when cycles with one transferred embryo were compared with cycles with two transferred embryos, the pregnancy, birth and twins rates were all significantly higher when two embryos were transferred, regardless of the day of embryo transfer. 

## 4. Discussion

The results of our study indicate that when there are only one or two embryos available and ET is performed on day five of preimplantation development (D5 ET group), this leads to a higher birth rate compared to cycles when ET is performed on day three (D3 ET group). While the pregnancy rate was similar between the groups, the difference in the live birth rate probably arose due to the non-significant higher miscarriage rate in D3 ET group. Furthermore, when we checked whether the different mean age of the women between the groups could be the reason for such an observation, it was revealed that when only patients aged 36 years or more were analyzed, the pregnancy rate, miscarriage rate and birth rate were very similar, despite a higher mean number of aspirated oocytes and obtained embryos in the D5 ET group. Furthermore, in the group of patients aged 35 years or less, we observed a weak trend for a higher live birth rate and a non-significant lower miscarriage rate in the D5 ET group. Therefore, we suggest that the higher live birth rate we observed in the D5 ET group when all patients were analyzed together arose due to the differences in the cycle outcome in the group of patients younger than 36 years. In addition, some other studies have suggested that the rate of live birth is higher if ET in performed on day five [8,9,10,11,12], and the miscarriage rate is lower [11,13,14], but it is not clear if the cumulative LBR is also higher [8]. In contrast, other studies have indicated that there is no difference in the pregnancy or live birth rates [3,15,16].

Our data contradict the data published in similar studies, although it must be emphasized that there are just a few similar studies. For instance, Haas et al. [17] retrospectively analyzed the data of 102 patients who had ET on day three compared to the data of 429 patients who had ET on day five, and all of these patients had at most two embryos obtained. The main conclusion of their study was that it is not important if the embryos are transferred on day three or day five because the cumulative pregnancy rate is similar in both cases. Unfortunately, their study had a lower number of included patients compared to ours and did not specifically determine the miscarriage and birth rates and, furthermore, part of their cycles was frozen-thawed. This is important because our data shows, similarly to theirs, that the pregnancy rate is similar regardless the day of transfer, but the live birth rate is higher after day five ET, probably due to the non-significant higher miscarriage rate after cleavage stage ET. Furthermore, the clinical pregnancy rate reported in the study by Haas et al. [17] was similar to our data. For instance, their pregnancy rate was 22% for ETs on day three and 24.6% for ETs on day 5five while our data showed 20.5% and 22.7% pregnancy rates per ET. Unfortunately, their study did not report the live birth rate. Another similar study by Xiao et al. [18] retrospectively studied cycles where there was only one viable embryo obtained. Their data suggest that biochemical pregnancy, clinical pregnancy and the live birth rate are significantly better when embryos are transferred at the cleavage stage compared to day four-six ET. This is again different to our conclusions, but care must be taken because their study included cycles where exactly one viable embryo was obtained on day three, and we included cycles with one-two embryos. When we checked only the subgroup of couples, where only one embryo was transferred, we did not find any significant difference in the pregnancy and birth rates. Furthermore, if we compare our data to Xiao et al.’s data, it seems their clinical pregnancy and live birth rates for day three ET (14.7% and 9.7%) and day four-six ET (6.8% and 4.4%) were lower than the pregnancy and live birth rates suggested by our data (day 3 ET (16.6% and 10.9%) and day 5 ET (19.5% and 14.4%). Interestingly, Xiao et al. [18] claimed that day three ETs were more likely to have confounding characteristics at the baseline associated with a poorer outcome. From this, it could be suggested that in cases of poor response, day three ET could be suggested; however, the study by Berkkanoglu et al. [19] indicated that this might not be the case. They compared their group of poor responders (4 or less oocytes collected) according to the day of ET. They concluded that the pregnancy and live birth rates per ET are significantly higher when day five ET is performed. On the contrary, Dirican et al. [7] suggest that the pregnancy rate is increased only for patients with six or more zygotes, while in patients with five or less zygotes, there is no difference between the blastocyst and cleavage-stage transfers. If we put our data in the context of the number of aspirated oocytes, on average, the patients included in our study had more than four aspirated oocytes, regardless of the day of ET, although the D5 ET group had a significantly higher number of retrieved oocytes. While the difference is still less than one retrieved oocyte, it should be noted that some studies indicate that there is positive association between the number of retrieved oocytes and the quality of day two/three embryos, day five embryos, and euploid embryos [20,21,22,23] and the cumulative live birth rate [24,25,26,27]. On the contrary, it was even shown that the rate of top-quality embryos is decreased by 0.5% for every oocyte obtained, but increased by 0.7% if the women’s age increased by 1 year [28]. One of the latest studies also showed that the live birth rate may even decline with a high number of retrieved oocytes, but this is only when over 25 oocytes are retrieved [29]. However, as expected, the live birth rate increased when 1–25 oocytes were retrieved and it was 17.2% in a subgroup of patients with 1–5 oocytes retrieved [29], which is similar to our data on day five ET (16.8%) and higher than our data for day three ET (13.8%) (73% of patients included in our study retrieved 5 or less oocytes). These data are also similar to another latest studies exploring correlation of number of retrieved oocytes with live birth rate, where 16.1% cumulative live birth rate was found for the group of patients with 0–5 oocytes retrieved [30]. On contrary to previously mentioned study [29], this study showed that the cumulative live birth rate also increased when over 20 oocytes were retrieved, but with diminishing returns [30]. In our case, the correlation analysis did not find any association between the number of retrieved oocytes and the pregnancy and birth rates. The reasons for the difference in the outcomes between day three and day five ET can be explained by the higher implantation potential that blastocysts have when compared to cleavage stage embryos, due to selection [2]. However, because, in the case of our study, there was no selection, or it was significantly limited due to only one or two embryos being obtained in the cycle, this probably cannot explain our results. Another explanation, more plausible to our case, is that it is not physiologically optimal to expose cleavage stage embryos to the uterine conditions because at this stage of development they should be still travelling through the fallopian tube [2]. For instance, oxygen tension decreases during this travelling and in the uterus, the environment is almost anoxic [31] and, furthermore, the embryo metabolism changes during development [32]. 

As this is a retrospective study, there are some limitations in our data. We captured data from 15 years of clinical work and, during this time, there were some important improvements introduced into clinical practice. For example, the culture media from different manufacturers were used and, furthermore, from 2015, there was a shift towards culturing embryos mostly in single step culture medium. Furthermore, from 2008 onwards, embryos were mostly cultured in a lower oxygen concentration (5%). As all of these changes applied to all of the patients treated at the same time period, there should not be any negative or positive effect applied to this specific group of patients. Another limitation of this study is that we cannot evaluate and show more detailed clinical characteristics of the patients (for instance hormone levels, BMI, etc.) because this is a long-term retrospective study, and we only have digital data for the last few years. 

To conclude, the most important message from our retrospective study is that the birth rate is significantly higher when ET is performed on day five instead of day three when there are only one or two embryos obtained in cycle.

## Figures and Tables

**Table 1:** Pearson correlation coefficients (r) between evaluated parameters for all cycles together are presented. A *p*-value of < 0.05 was considered statistically significant (* *p* < 0.05; ** *p* < 0.01; *** *p* < 0.001).

	Age of Patients	Gonado-Trophines Dose	Number of Aspirated Oocytes	Number of Immature Oocytes	Number of Embryos	Number of Transferred Embryos	Day of Embryo-Transfer	Pregnancy	Births	Number of Born Children
Age of patients	1									
Gonado-trophines dose	0.258 ***	1								
Number of aspirated oocytes	−0.235 **	−0.087 ***	1							
Number of immature oocytes	−0.155 ***	−0.092 ***	0.715 ***	1						
Number of embryos	−0.070 ***	−0.028	0.247 ***	0.045 *	1					
Number of transferred embryos	0.035	0.011	0.152 ***	−0.011	0.788 ***	1				
Day of embryo-transfer	−0.086 ***	−0.047 *	0.070 ***	0.059 **	0.122 ***	−0.008	1			
Pregnancy	−0.107 ***	−0.064 **	−0.009	−0.035	0.112 ***	0.110 ***	0.024	1		
Births	−0.139 ***	−0.069 ***	−0.007	−0.047 *	0.091 ***	0.092 ***	0.039 *	0.799 ***	1	
Number of born children	−0.075	0.053	0.047	−0.088	0.203 ***	0.273 ***	−0.008	0.016	0.016	1

**Table 2:** Pearson correlation coefficients (r) between evaluated parameters only for cycles with singletons born. A *p*-value of < 0.05 was considered statistically significant (* *p* < 0.05; ** *p* < 0.01; *** *p* < 0.001).

	Age of Patients	Gonado- Trophines Dose	Number of Aspirated Oocytes	Number of Immature Oocytes	Number of Embryos	Number of Transferred Embryos	Day of Embryo- Transfer	Gestational Age	Birth Weight
Age of patients	1								
Gonado- trophines dose	0.277 ***	1							
Number of aspirated oocytes	−0.066	−0.044	1						
Number of immature oocytes	−0.040	−0.092	0.548 ***	1					
Number of embryos	−0.021	0.062	0.327 ***	0.059	1				
Number of transferred embryos	0.114 *	0.063	0.237 ***	0.004	0.763 ***	1			
Day of embryo- transfer	−0.115 *	−0.056	0.152 **	0.140 **	0.124 *	−0.038	1		
Gestational age	0.020	−0.029	0.067	−0.005	0.084	0.079	−0.059	1	
Birth weight	0.070	−0.017	0.059	−0.019	0.045	0.072	−0.018	0.782 ***	1

**Table 3:** Basic characteristics of patients included into study. Significant differences are marked with * (*p* < 0.05).

	Day 3 ET Group	Day 5 ET Group	*p*-Value
Number of cycles/ETs	1927	731	
Mean age of patients (±SD)	36.0 ±4.4	35.1 ±4.5	<0.001 *
Tubal factor of infertility	97 (5.0%)	34 (4.7%)	0.680
Endometriosis	86 (4.5%)	22 (3.0%)	0.090
Endocrine factor of infertility	63 (3.3%)	20 (2.7%)	0.480
Uterine causes of infertility	98 (5.1%)	41 (5.6%)	0.590
Cervical causes of infertility	14 (0.7%)	3 (0.4%)	0.361
Combined factors of female infertility	218 (11.3%)	75 (10.3%)	0.439
Male and female factor of infertility	766 (39.8%)	330 (45.1%)	0.012 *
Male factor of infertility	453 (23.5%)	150 (20.5%)	0.100
Unexplained infertility	132 (6.9%)	56 (7.7%)	0.467
Mean gonadotrophine dose in stimulated cycles (±SD)	2228 ± 911	2133 ± 860	0.015 *

**Table 4:** The outcome of cycles with only 1 or 2 embryos available for ET. Significant differences are marked with * (*p* < 0.05).

	Day 3 ET Group	Day 5 ET Group	*p*-Value
Number of cycles/ETs	1927	731	
Number of aspirated oocytes (mean number ± SD)	8285 (4.3 ± 3.2)	3503 (4.8 ± 2.8)	<0.001 *
Number of immature oocytes (mean number ± SD)	1877 (1.0 ± 1.6)	870 (1.2 ± 1.7)	0.002 *
Number of polyploidies (mean number ± SD)	355 (0.2 ± 0.5)	165 (0.2 ± 0.6)	0.080
Number of zygotes	3047	1265	
Number of embryos (mean number ± SD)	2869 (1.5 ± 0.5)	1188 (1.6 ± 0.5)	<0.001 *
Mean number of transferred embryos	1.4 ± 0.5	1.4 ± 0.5	0.680
Number of pregnancies (% per ET)	395 (20.5%)	166 (22.7%)	0.212
Miscarriages per pregnancy	125 (31.6%)	41 (24.7%)	0.100
Ectopic pregnancies	5 (1.3%)	2 (1.2%)	1
Births	265 (13.8%)	123 (16.8%)	0.045 *
Twins	25 (9.4%)	11 (8.9%)	0.888
Mean gestational age (including twins’ births)	38.7 ± 2.8	38.3 ± 2.7	0.224
Mean gestational age for singletons	38.9 ± 2.6	38.6 ± 2.6	0.272
Mean birthweight of singletons (g)	3217 ± 644	3191 ± 680	0.737

**Table 5:** The main outcome of cycles according to the age of patients and the day of embryo transfer. Significant differences are marked with * (*p* < 0.05).

	≤35 Years Old			≥36 Years Old		
	Day 3 ET Group	Day 5 ET Group	*p*-Value	Day 3 ET Group	Day 5 ET Group	*p*-Value
Number of cycles	843	381		1084	350	
Mean age of patients (±SD)	31.8 ± 2.7	31.5 ± 2.8	0.168	39.2 ± 2.2	39.0 ± 2.2	0.089
Mean number of aspirated oocytes	5.0 ± 3.8	5.4 ± 3.0	0.059	3.8 ± 2.6	4.1 ± 2.4	0.012 *
Mean number of immature oocytes	1.3 ± 1.9	1.4 ± 1.9	0.202	0.8 ± 1.3	1.0 ± 1.3	0.013 *
Mean number of embryos	1.5 ± 0.5	1.6 ± 0.5	<0.001 *	1.5 ± 0.5	1.6 ± 0.5	<0.001 *
Mean number of transferred embryos	1.4 ± 0.5	1.4 ± 0.5	0.780	1.4 ± 0.5	1.4 ± 0.5	0.502
Pregnancy rate	205 (24.3%)	105 (27.6%)	0.227	190 (17.5%)	61 (17.4%)	1
Miscarriages per pregnancies	49 (23.9%)	18 (17.1%)	0.171	76 (40.0%)	23 (37.7%)	0.751
Births per ETs	152 (18.0%)	85 (22.3%)	0.079	113 (10.4%)	38 (10.9%)	0.823
Twins	14 (9.2%)	9 (10.6%)	0.729	11 (9.7%)	2 (5.3%)	0.518

**Table 6:** The main outcomes of cycles according to the number of embryos transferred into uterus and the day of embryo transfer. Significant differences are marked with * (*p* < 0.05).

		Day 3 ET Group	Day 5 ET Group	*p*-Value
Number of cycles/ETs	ET of 1 embryo	1135	437	
	ET of 2 embryos	792	294	
Mean age of patients (±SD)	ET of 1 embryo	35.8 ± 4.5	35.1 ± 4.6	0.005 *
	ET of 2 embryos	36.2 ± 2.4	35.2 ± 4.4	<0.001 *
	*p*-value	0.045 *	0.839	
Number of aspirated oocytes (mean number ± SD)	ET of 1 embryo	3.9 ± 3.3	4.5 ± 2.9	0.001 *
	ET of 2 embryos	4.9 ± 3.1	5.3 ± 2.7	0.093
	*p*-value	<0.001 *	<0.001 *	
Number of pregnancies (% per ET)	ET of 1 embryo	188 (16.6%)	85 (19.5%)	0.176
	ET of 2 embryos	207 (26.1%)	81 (27.6%)	0.639
	*p*-value	<0.001 *	0.010 *	
Miscarriages per pregnancy	ET of 1 embryo	61 (32.4%)	21 (24.7%)	0.196
	ET of 2 embryos	64 (30.9%)	20 (24.7%)	0.296
	*p*-value	0.740	1	
Births	ET of 1 embryo	124 (10.9%)	63 (14.4%)	0.055
	ET of 2 embryos	141 (17.8%)	60 (20.4%)	0.327
	*p*-value	<0.001 *	0.034 *	
Twins	ET of 1 embryo	1 (0.8%)	1 (1.6%)	1
	ET of 2 embryos	24 (17.0%)	10 (16.7%)	1
	*p*-value	<0.001 *	0.003 *	

## Data Availability

The data presented in this study are available on request from the corresponding author.
